# Recurrence of hepatocellular carcinoma following deceased donor liver transplantation: case series

**DOI:** 10.20517/2394-5079.2019.51

**Published:** 2020-03-20

**Authors:** Cem Simsek, Amy Kim, Michelle Ma, Nilay Danis, Merve Gurakar, Andrew M. Cameron, Benjamin Philosophe, Jacqueline Garonzik-Wang, Shane Ottmann, Ahmet Gurakar, Behnam Saberi

**Affiliations:** 1 Johns Hopkins University School of Medicine, Division of Gastroenterology and Hepatology-Transplant Hepatology, Baltimore, MD 21205, USA.; 2 Johns Hopkins University Bloomberg School of Public Health, Baltimore, MD 21205, USA.; 3 Johns Hopkins University School of Medicine, Division of Transplant Surgery, Baltimore, MD 21205, USA.

**Keywords:** Hepatocellular carcinoma, liver transplant, liver resection, locoregional therapy

## Abstract

**Aim:**

We aimed to study the clinical and pathological characteristics of liver transplant recipients with hepatocellular carcinoma recurrence.

**Methods:**

We reviewed the data for 26 patients who had tumor recurrence after deceased donor liver transplant for hepatocellular carcinoma at the Johns Hopkins Hospital from January 2005 to December 2015.

**Results:**

In total, 88% of recipients were males. The mean age was 59 years. On explant, poor differentiation was detected in 43%, while 73% had microvascular invasion. Overall, 62% were diagnosed to be outside of Milan criteria. Out of these, 15% met the criteria for downstaging. Twenty (77%) patients had pre-transplant alpha fetoprotein levels ≥ 20 ng/mL. In 54% of patients, the location of hepatocellular carcinoma (HCC) recurrence was extrahepatic, followed by intrahepatic in 31% and both intra- and extrahepatic in 15%. The post-transplant tumor recurrence was diagnosed at a mean of 427 days (range 34–1502). Fifty percent of HCC recurrences were diagnosed within one year following liver transplant. Twenty (77%) patients received treatment for their recurrent HCC: external radiation (*n* = 10), surgical resections (*n* = 8; brain 4, spine 2, bone 1, and Whipple surgery 1), sorafenib (*n* = 7), locoregional therapy (*n* = 5). Overall, 24 out of 26 (92%) recipients died within four years after the transplant.

**Conclusion:**

HCC recurrence after liver transplant is infrequent. More than fifty percent of HCC recurrences following liver transplant are extrahepatic. Despite better recipient selection for liver transplant, the curative options are limited in recurrent cases and associated with extremely poor outcomes.

## INTRODUCTION

Liver transplant (LT) has become the treatment of choice in patients with hepatocellular carcinoma (HCC) and cirrhosis who meet the Milan criteria (MC)^[1]^. Although additional extended criteria models have been proposed, HCC recurrence following LT remains an unfortunate incident associated with poor survival^[2,3]^. Tumor biology and alpha fetoprotein (AFP), as well as tumor size and number, have been proposed by various groups as other potentially relevant factors of tumor recurrence^[4–6]^.

Overall, two thirds (2/3) of patients, who develop recurrent HCC post-LT, present with extrahepatic recurrence^[7,8]^. The treatment of choice in post LT HCC recurrence is determined based on the site and the extent of the recurrence^[8]^. However, treatments are not standardized and mostly based on expert opinion and retrospective studies^[9]^. Surgical treatment options have been proposed with promising outcomes in selected patients^[10,11]^. Locoregional therapy options, transarterial chemoembolization, radiofrequency ablation, and stereotactic radiation are considered in selected cases^[9]^.

In a recent report, we published our experience in LT recipients with HCC at the Johns Hopkins University Comprehensive Liver Transplant Center^[12]^. As a follow-up study, we aimed to study the clinicopathological features and outcomes of 26 cases with HCC recurrence following LT. In addition, we evaluated the details on the outcomes and the application of different treatment modalities in this group.

## METHODS

The study was approved by the institutional review board at the Johns Hopkins Hospital. HCC-related deceased donor LT recipients between January 2005 and December 2015 were evaluated. In total, 26 patients with post-LT HCC recurrence were identified among 165 recipients who were included in the study. All recipients were listed following a standard work up and discussion at the weekly selection meeting. They were within Milan criteria or downstaged into Milan criteria. The transplant was performed by piggyback technique. Postoperative HCC surveillance consisted of contrasted cross-sectional imaging with computerized tomography or magnetic resonance imaging with AFP every three months for the first year and every six months for the second and third years. There was no set therapeutic protocol for recurrence; treatment options were discussed in a multidisciplinary fashion. The Pre-LT AFP was obtained within the past three months prior to deceased donor liver transplantation (DDLT) and immediate post-LT AFP was obtained within three months post DDLT.

Data on clinical, radiologic, pathology, HCC recurrence, and survival were collected from the records, reviewed, and analyzed. Explant pathologies were reviewed retrospectively, and the following tumor parameters were collected: size, number of lesions, microvascular invasion status, and differentiation. It was determined whether patients met the Milan or University of California San Francisco (UCSF) criteria based on the number and size of HCC lesions on explant pathology. The data collected for categorical variables were reported as percentages. Data for continuous variables were reported by the mean and standard deviation. Patient survivals were analyzed using Kaplan-Meier statistics. STATA V.13 (StataCorp college station, TX) was used to perform the statistical analyses.

## RESULTS

### Patient characteristics

**Figure 1.**
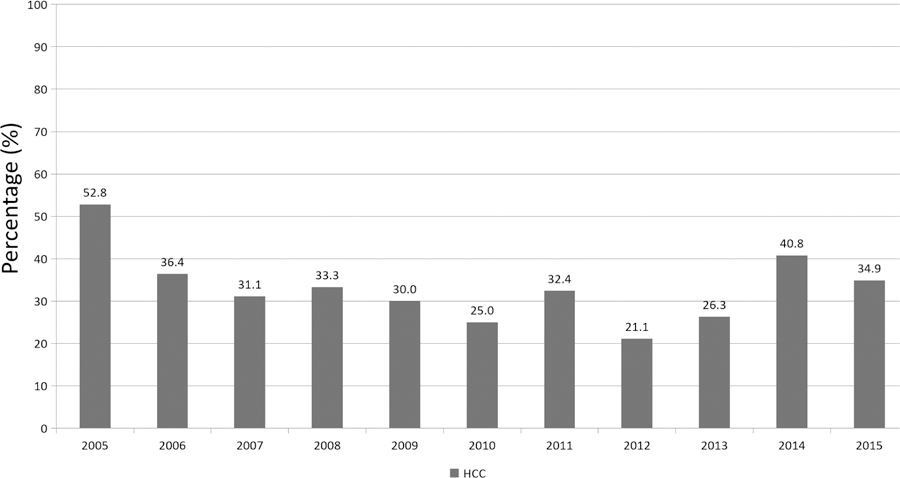
Overall, rate of deceased donor liver transplant for hepatocellular carcinoma indication at the Johns Hopkins Hospital from 2005 to 2015. HCC: hepatocellular carcinoma

Among the deceased donor LT recipients, HCC was the primary indication for transplantation varying from 21% to 53% of patients [Figure 1] according to the year. Clinical information on the 26 LT recipients with recurrent HCC is summarized in Table 1. Patients were predominantly male (88.5%) with a mean age of 59 years (range 47–72 years). The majority of recipients were white (*n* = 17, 65.4%), followed by African American (*n* = 7, 27.0%) and Asian (*n* = 2, 7.6%) ethnicities. Primary etiology of liver disease was chronic hepatitis C (positive hepatitis C antibody and/or hepatitis C RNA) in 13 patients (50%) and hepatitis C and alcoholic liver disease in 6 (23%) patients. Chronic hepatitis B (positive hepatitis B surface antigen and/or hepatitis B DNA) was seen in three patients (11.5%), followed by alcoholic liver disease (*n* = 2, 7.7%), and non-alcoholic fatty liver disease (*n* = 1, 3.9%).

### Laboratory results

The average model for end-stage liver disease (MELD) score was 13, ranging from 6 to 35. Mean AFP was 27.6 ng/mL for pre-LT *vs.* 23.6 ng/mL for post-LT time periods [Tables 1 and 2]. Four patients had pre-LT AFP levels of > 1000 ng/mL. The other available laboratory results are summarized in Table 1.

### Immunosuppression

Overall, nine (34.6%) patients were treated with mammalian target of rapamycin (mTOR) treatment with sirolimus in eight and everolimus in one patient. Seventeen patients received Tacrolimus-based therapy.

### Explant-pathology findings

In the explant pathologies of LT recipients, 9 (34.6%) patients had only one lesion and 11 (42.4%) had 4 or more lesions. The average for the largest lesion size was 4.3 cm. In total, 12 patients (46.1%) had multi-lobar tumors and 13 (50%) had tumors that were located in the right lobe. Overall, 10 patients (38.4%) were within MC criteria and 11 patients (42.3%) were within UCSF criteria. Four patients (15.4%) were downstaged to MC with locoregional treatment. Seventeen (65.4%) patients underwent locoregional therapy before transplant. None of the tumors were well-differentiated. Overall, 14 (53.8%) patients had moderately differentiated HCC. Eleven (42.3%) patients had HCC with poor differentiation. Microvascular invasion was detected in 19 of the 26 cases (73.1%) while one patient had bile duct invasion only.

### Recurrence and survival

**Figure 2.**
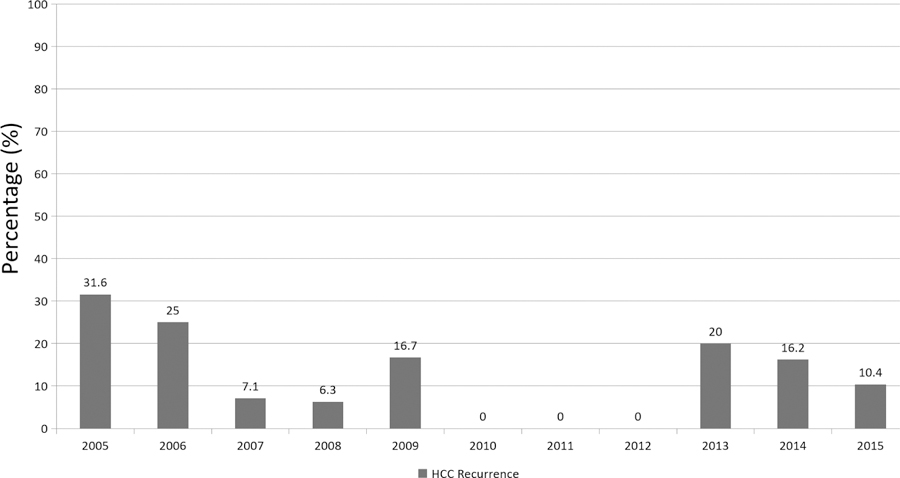
Overall, per year rate of hepatocellular carcinoma recurrence in deceased donor liver transplant recipients at the Johns Hopkins Hospital from 2005 to 2015. HCC: hepatocellular carcinoma

The overall rate of HCC recurrence following LT in our series was 15%. The rate of HCC recurrence has improved over the years with a recurrence rate of 10% in 2015 [Figure 2]. Mean time for diagnosis of HCC recurrence after LT was 427 days, ranging from 34 to 1502 days. The site of HCC recurrence was intrahepatic in 8 (31%), extrahepatic in 14 (54%), and both intra- and extrahepatic in 4 (15%) patients. Overall, 31% of recipients had intrahepatic HCC recurrence following LT when compared to 69% with extrahepatic recurrence. Twenty-two percent of the patients who had extrahepatic involvement had concomitant liver involvement. The most common sites of extrahepatic involvement were the lungs (44.4%) and bones (44.4%) (spine, rib, pelvis, and humerus), followed by mediastinum (27.8%), brain (22.2%), portal lymph nodes (11.1%), gastro-hepatic ligament (5.6%), adrenal gland (5.6%), pleura (5.6%), and peritoneum (5.6%).

**Figure 3.**
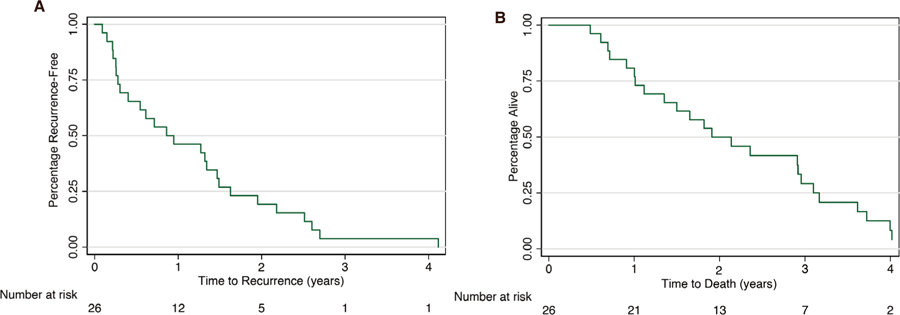
Survival analysis for 26 liver transplant recipients with hepatocellular carcinoma recurrence: A: Kaplan-Meier curve for recurrence-free survival; B: Kaplan-Meier curve for overall survival

A range of different treatment modalities was used for recurrences [Table 3]. Six (21.4%) of the 26 patients were managed with supportive care. The remaining 20 cases received various treatment modalities including locoregional therapy (transarterial chemoembolization in 3, Y 90 in 1, and radiofrequency ablation in 1), external radiation in 10, and surgical resections in 8 (brain 4, spine 2, bone 1, and Whipple surgery in 1). Nine (32%) patients received combination therapies of the above-mentioned modalities. Seven patients (27%) received sorafenib. An additional two patients received chemotherapy regimens other than sorafenib [Table 3]. Recurrence-free survival and overall survival are shown in Figure 3. Patients who developed HCC recurrence following LT had an extremely poor overall survival (7.7%). In total, 19% of patients died within one year following LT. Overall, 24 out of 26 (92.3%) patients died throughout the four-year follow-up period. Timing of death relevant to the time of LT is shown in Table 3.

## DISCUSSION

In this series, we report a rate of 15% HCC recurrence following deceased donor LT at our transplant program. This rate is consistent with the literature report of 15%−20% post-LT HCC recurrence^[13]^. It is well known that the patients who are outside of MC prior to LT have higher rates of tumor recurrence following LT, compared to those within the MC^[1]^. Although all of the patients within our series were thought to be within MC radiographically prior to LT, according to radiology findings, only 34% were within the criteria by reviewing the explant. When including an additional four (15%) patients who were downstaged, in total 49% were within MC based on pathology. This discrepancy between radiology and pathology has been previously described by other groups in the literature^[13]^.

Our sites of recurrence findings are very similar to the recent reports^[8]^. In a systematic review of post-LT HCC recurrence, extrahepatic site was the most common site of recurrence in 67% of cases, compared to intrahepatic in 33%^[8]^. The extrahepatic sites of involvement included: bone, pulmonary, adrenal, lymph nodes, and brain^[8]^.

Within our series, 54% of the HCC recurrences were diagnosed within 1 year post-OLT, while 81% and 96% of recurrences occurred within 2 and 3 years following OLT, respectively. The average time to HCC recurrence within our series was 427 days (range 34–1502 days). It is shown by others that early versus late recurrence is a predictor of post-LT survival^[14]^. The patients with early HCC recurrence, defined as recurrence within 24 months post-LT, have a worse prognosis^[14]^. There are a few potential theories for early HCC recurrence post-LT: (1) biologically rapid growing, aggressive tumors; (2) lack of high-quality pre-LT imaging or overlooking intra- or extrahepatic imaging^[8]^; (3) extrahepatic microscopic viable HCC cells that could not be detected by conventional imaging prior to LT; and (4) presence of circulating tumor cells that seed to other sites. The mechanism by which the late recurrence occurs is unclear^[15]^. Presence of pre-LT HCCs that are biologically slow growing, or development of *de novo* HCC recurrence in the liver allograft could be the cause. Within our series, we did not have any cases who had HCC recurrence that occurred or were diagnosed beyond five years following LT.

The selection of an ideal treatment for post LT HCC recurrence is a matter of debate, and the evidence is mainly based on expert opinion and non-randomized cohort studies^[9]^. The treatment modality will vary based on the type of recurrence (intrahepatic versus extrahepatic), organ of involvement, and extent of involvement. This includes a wide range of surgical (intra- or extrahepatic resection and re-transplantation) and non-surgical treatments (locoregional therapies, sorafenib, other systemic chemotherapy, mTOR inhibitors, and best supportive care)^[16]^.

Surgical options including extrahepatic resection, liver graft resection, and liver re-transplant have also been considered for patients presenting with HCC recurrence. In 2004, the Mount Sinai group reported resection of the liver allograft in five out of 18 recipients with HCC recurrence^[11]^. The authors concluded that, in selected cases with recurrent intrahepatic-HCC, liver resection improved survival^[11]^ Similarly, Kornberg *et al.*^[10]^ reported that HCC recurrence should be treated surgically in eligible patients with good long-term outcomes. In multivariate analysis of post-LT HCC recurrence, late tumor recurrence (> 24 months) and surgical resection were the two independent predictors of survival^[10]^. A systematic review in 2015 reported that the surgical approach to localized intra- or extra-hepatic recurrences are uneventful and not associated with higher mortality^[8]^. Retransplantation for recurrent HCC is not a practical option^[17]^ due to the higher risk of recurrence with a limited organ availability.

Sorafenib, a multikinase inhibitor, has been approved as first-line treatment for the management of advanced-stage HCC following two clinical trials in 2008 and 2009^[18,19]^. In a multicenter phase 2, blinded placebo-controlled, clinical trial, the efficacy of sorafenib for preventing HCC recurrence post-LT in high-risk recipients is being actively investigated [ClinicalTrials.gov identifier (NCT number): NCT01624285]. There are currently no systemic therapies that have been shown to improve survival in HCC recurrence post-LT. Recently, other tyrosine kinase inhibitors were approved as first- or second-line treatment in HCC in the non-transplant setting^[20]^. The role of these agents as adjuvant therapy or post-LT HCC recurrence is unclear and deserves further investigation in the near future. Nivolumab, an anti-PD1 inhibitor, was recently approved for the treatment of HCC, as second line, in the non-transplant setting, with the objective response rate of 20%^[21]^. The role of immunotherapy among post-LT recipients with HCC has not been yet established. It is possible that the immunotherapy will affect the liver allograft leading to acute cellular rejection^[22]^.

Mammalian target of rapamycin (mTOR), a serine/threonine protein kinase, has been shown to be upregulated in 40%−50% of HCCs. mTOR is involved in the regulation of cell metabolism and growth^[23]^. Therefore, various studies have suggested that mTOR inhibitors may have antineoplastic properties in HCC patients and mTOR inhibitors should be used after LT. In a meta-analysis of 2950 patients from five studies, sirolimus-based immunosuppression reduced the rate of tumor recurrence and improved overall survival^[24]^.

HCC recurrence following LT is an unfortunate event and associated with poor outcomes. In a recent meta-analysis, the median overall survival was 13 months following the diagnosis of HCC recurrence post-LT^[8]^. Herein, supportive care was associated with the lowest survival rate of 3.3 months^[8]^. There is no standardized protocol regarding the type and frequency of post-LT cross-sectional imaging in surveillance of HCC LT recipients. It is important to note that more than 50% of patients develop tumor recurrences that are outside of liver (extrahepatic), therefore imaging limited to the liver may not be sufficient for the diagnosis of majority of HCC recurrences. We also note that AFP is a useful marker in post-LT HCC surveillance only for high-AFP-secreting tumors. Four patients in our study had pre-AFP levels of > 1000 ng/mL. It is well known that patients with high AFP producing tumors have worse tumor biology and have worse outcomes^[12,25]^. HCC candidates need to have AFP of ≤ 1000 ng/mL to receive extra points to shorten the waiting period for liver transplantation^[25]^. The overall prognosis of HCC recurrence following LT is poor in the majority of cases and there are no available studies evaluating cost-effectiveness of surveillance protocols specific to this group of patients.

In conclusion, HCC recurrence post liver transplant is an unfortunate event associated with extremely poor survival. The majority of the cases are early recurrence occurring 1–2 years following liver transplantation. More than 50% of HCC recurrences are extrahepatic. Therefore, post-liver transplant imaging confined to the liver may not be enough to detect all of the recurrences. In patients with AFP producing tumors, this marker may also be helpful to diagnose the HCC recurrence. There is no general consensus on the treatment for post liver transplant hepatocellular carcinoma recurrence. The current reports are mainly based on single-center retrospective experience.

## Figures and Tables

**Table 1.** Characteristics of the study population

Variable	*n* = 26
Clinical features	
Male sex, *n* (%)	23 (88.5%)
Age (years)	58.9 (6.8)
Ethnicity, *n* (%)	
White	17 (65.4%)
African American	7 (27.0%)
Asian	2 (7.6%)
Etiology	
HCV	13 (50%)
HBV	3 (11.5%)
ALD	2 (7.7%)
NAFLD	1 (3.9%)
HCV/ALD	6 (23%)
Other	1 (3.9%)
Explant pathology	
Number of lesions, *n* (%)	
1	9 (34.6%)
2	3 (11.5%)
3	3 (11.5%)
> 4	11 (42.4%)
Largest lesion (cm)	4.3 (3.8)
Tumor location, *n* (%)	
Right lobe	13 (50%)
Left lobe	1 (3.9%)
Multi-lobar	12 (46.1%)
Tumor differentiation, *n* (%)	
Well	0 (0%)
Moderate	14 (53.8%)
Poor	11 (42.3%)
Unknown	1 (3.9%)
Microvascular invasion, *n* (%)	
Yes	19 (73.1%)
No	6 (23%)
Bile duct invasion	1 (3.9%)
Total number of loco-regional therapies, *n* (%)	
0	9 (34.6%)
1	9 (34.6%)
2	5 (19.2%)
> 2	3 (11.6%)
Patients with viable tumor, *n* (%)	
Yes	25 (96.2%)
No	1 (3.8%)
Within Milan, *n* (%)	
Yes	10 (38.4%)
No	16 (61.6%)
Downstaged to Milan, *n* (%)	4 (15.4%)
Within UCSF, *n* (%)	
Yes	11 (42.3%)
No	15 (57.7%)
Downstaged to UCSF, *n* (%)	3 (11.5%)
Laboratory	
Pre-LT AFP (ng/mL)	27,578 (133,183)
Post-LT AFP (ng/mL)	23,586 (81,707)
MELD	13 (7)
WBC (10^9^/L)	6 (2.2)
Hgb (g/dL)	12.9 (2.7)
MCV (fL)	91 (6)
PLT (10^3^/µL)	116 (67)
BUN (mg/dL)	15 (6)
Creatinine (mg/dL)	1.1 (0.6)
TP (g/dL)	7.2 (0.8)
Alb (g/dL)	3.6 (0.7)
ALP (U/L)	141 (58)
AST (U/L)	109 (167)
ALT (U/L)	71 (122)
T.Bili (mg/dL)	2.2 (2.4)
PT (sec)	14 (4.1)
INR	1.3 (0.4)

Clinical and pathological characteristics of the 26 recipients with hepatocellular carcinoma recurrence following liver transplant. Quantitative data are expressed as mean and categorical variables are reported as percentages. AFP: alpha fetoprotein; ALD: alcoholic liver disease; Alb: albumin; ALP: alkaline phosphatase; AST: aspartate aminotransferase; ALT: alanine aminotransferase; BUN: blood urea nitrogen; HBV: hepatitis B virus; HCV: hepatitis C virus; Hgb: hemoglobin; INR: international normalized ratio; LT: liver transplant; MCV: mean corpuscular volume; MELD: model for end-stage liver disease; NAFLD: non-alcoholic fatty liver disease; PLT: platelet count; PT: prothrombin time; TP: total protein; T.Bili: total bilirubin; UCSF: University of California San Francisco; WBC: white blood cell count

**Table 2.** Alpha fetoprotein levels pre and post-liver transplant

Patient	Pre-LT AFP	Initial post-LT AFP	AFP at recurrence
1	9	7	2019
2	28,139	365,210	NA
3	135	4.1	15
4	3.6	0.6	1.7
5	27	3864	NA
6	488	57	86
7	22	2	26
8	23	12	1416
9	162	6.4	7
10	169	682	3342
11	34	3	389
12	48	4	12
13	323	76	157
14	7	21	NA
15	23	10	51
16	4659	25,154	NA
17	304	35	5.4
18	3.3	4.3	210
19	1707	100	47,304
20	34	2	3.7
21	680,000	217,576	120,848
22	22	2	17
23	4	2	NA
24	207	317	40.9
25	486	104	3677
26	5.2	5.5	4

Alpha fetoprotein levels (ng/mL) pre- and post-liver transplant in 26 liver transplant recipients with hepatocellular carcinoma recurrence. AFP: alpha fetoprotein; LT: liver transplant; NA: data not available

**Table 3.** Specific characteristics of the tumors, treatment, and outcomes

Patient	Number of lesions	Largest lesion (cm)	Within Milan criteria	Downstaged	MVI	Differentiation	Diagnosis of recurrence following LT (in days)	Site of recurrence	Survival	Time of death following LT (days)	Treatment of recurrence
1	5	4	No	No	Yes	Moderate	984	Gastro-hepatic ligament, mediastinal	Died	1466	Sorafenib
2	1	8	No	Yes	Yes	Poor	314	Liver	Died	369	Supportive care
3	5	8.5	No	Yes	Yes	Moderate	950	Brain	Died	1062	Brain metastasis resection
4	Infiltrative	1.1	No	No	Yes	Moderate	536	Liver	Died	780	Y90
5	1	1	Yes	No	No	Moderate	80	Porta-hepatis	Died	366	Chemotherapy with capecitabine
6	1	7.5	No	No	Yes	Poor	102	Perihilar, lung	Died	222	Supportive care
7	Infiltrative	2	No	No	Yes	Poor	224	Brain, liver, adrenal	Died	1459	Brain metastasis resection and radiation, cryoablation of adrenal metastasis, Y90 in liver
8	3	1	Yes	No	No	Moderate	490	Humerus, brain	Died	663	Bone resection and radiation, brain met resection
9	8	4.3	No	No	Yes	Poor	291	Ribs, spine	Died	602	Sorafenib Spine surgery and radiation
10	2	1.5	Yes	No	No	Moderate	94	Portal nodes, mediastinal, lung	Died	253	Sorafenib, external radiation
11	2	1.6	Yes	No	Yes	Poor	464	Bone, lung	Died	547	Supportive care
12	1	5	Yes	No	Yes (bile duct)	Poor	482	Bile ducts	Died	1131	Whipple surgery, Sorafenib
13	5	6	No	No	Yes	Moderate	111	Lung, peritoneal carcinomatosis	Died	332	Supportive care
14	Infiltrative	2.8	No	No	Yes	Moderate	544	Pelvic bone	Died	1079	External radiation, Sorafenib
15	1	2.3	Yes	No	No	Moderate	795	Spine	Died	1319	Spine surgery and radiation
16	1	7.2	No	No	Yes	Poor	93	Pelvic bone, lung	Died	493	External radiation
17	5	3.5	No	No	Yes	Poor	261	Liver	Alive	-	TACE
18	3	3.5	No	Yes	Yes	Moderate	713	Lung, liver	Died	861	TACE
19	1	2.9	Yes	No	Yes	Poor	346	Liver	Died	407	Supportive care
20	1	2.8	Yes	No	Yes	Moderate	1502	Liver	Alive	-	RFA, TACE, Sorafenib
21	Infiltrative	19	No	No	Yes	Poor	78	Lung, bone, liver	Died	178	Chemotherapy with Gemcitabine/Cisplatin, external radiation
22	2	2.6	Yes	No	Yes	Poor	199	Liver	Died	260	Supportive care
23	5	2	No	No	No	Moderate	594	Brain	Died	1156	Brain metastasis resection
24	3	7.5	No	Yes	Yes	Moderate	147	Mediastinal lymph nodes, pleura	Died	697	Sorafenib
25	Infiltrative	4	No	No	Yes	Moderate	34	Spine, mediastinal	Died	1065	Spine radiation
26	1	2.2	Yes	No	No	No viable tissue	917	Lung, mediastinal	Died	1359	External radiation

Specific characteristics of the tumors, treatment, and outcomes in 26 liver transplant recipients with a recurrence of hepatocellular carcinoma following liver transplant. LT: liver transplant; MVI: microvascular invasion; RFA: radiofrequency ablation; TACE: transarterial chemoembolization
